# Public interest in benzoyl peroxide: A Google Trends analysis

**DOI:** 10.1111/jocd.16408

**Published:** 2024-06-14

**Authors:** Isabella J. Tan, Bianca Sanabria, Jimmy Dhillon, Babar Rao

**Affiliations:** ^1^ Rutgers Robert Wood Johnson Medical School New Brunswick New Jersey USA; ^2^ Center for Dermatology Rutgers Robert Wood Johnson Medical School Somerset New Jersey USA; ^3^ Department of Dermatology Weill Cornell Medicine New York New York USA

To the Editor,

In March 2024, Valisure petitioned the US FDA for the recall of benzoyl peroxide (BPO) products due to elevated benzene levels, raising global safety concerns.^1^ Amidst ongoing discussions within the dermatology community, understanding public interest in BPO becomes crucial. Google Trends data have emerged as a powerful tool for analyzing population interests, including healthcare trends.^2^ Google Trends is valuable for public health surveillance, spanning from monitoring consumer behavior to tracking emerging health concerns. Our study evaluates public interest in benzoyl peroxide post‐Valisure petition, offering insights that may help dermatologists make informed treatment decisions amidst safety concerns. Google Trends also offers dermatologists real‐time insights into patient concerns and emerging dermatological trends.^3^ By analyzing search patterns, dermatologists can proactively communicate with patients, adapt treatment plans, and stay responsive to evolving needs and expectations.

We conducted a Google Trends query to retrieve relative search volume (RSV) data. RSV values range from 0 to 100, with 100 representing the maximum popularity of the search term.^4^ Our investigation assessed public interest in BPO post‐Valisure petition by comparing RSV data for search terms from March–April 2024 with the corresponding periods in the preceding 3 years (2020–2023).

Search terms encompassing all BPO product names in the United States were selected based on benzoyl peroxide drug information from UpToDate to ensure comprehensive coverage of BPO‐related searches with available RSV data from 2020 to 2024.^5^ Statistical analyses were performed using SPSS 29.0.2.0, with *p*‐values less than 0.05 considered statistically significant.

Benzoyl peroxide exhibited a substantial decline in interest post‐petition, with the RSV decreasing from 86.19 in 2023 to 22.06 in 2024 (*p* < 0.001) (Table 1). This pattern was consistent across earlier years, indicating a sustained downward trend (Figure 1). Conversely, benzoyl peroxide wash demonstrated variable patterns, with a decline in RSV in 2024 compared to the previous year (*p* = 0.015) and a significant increase in 2022 (*p* < 0.001). Further analysis revealed consistent decreases in RSV for BPO products from 2023 to 2024 (*p* < 0.001), reflecting diminishing public interest post‐recall. Inova maintained stability in search volume, while PanOxyl experienced a significant decrease in RSV in 2024 compared to the preceding year (*p* < 0.001).

**TABLE 1:** Relative search volume of search terms.

Relative search volume (Avg)					
	Benzoyl peroxide	Benzoyl peroxide wash	BPO	Inova	PanOxyl
March 2024	22.06	40.31	28.59	71.38	46.59
March 2023	86.19	56.09	70.53	73.13	74.00
March 2022	79.47	70.19	60.09	79.53	64.22
March 2021	82.91	46.19	70.16	77.69	76.94
March 2020	78.00	37.47	71.38	73.75	32.84

**FIGURE 1:**
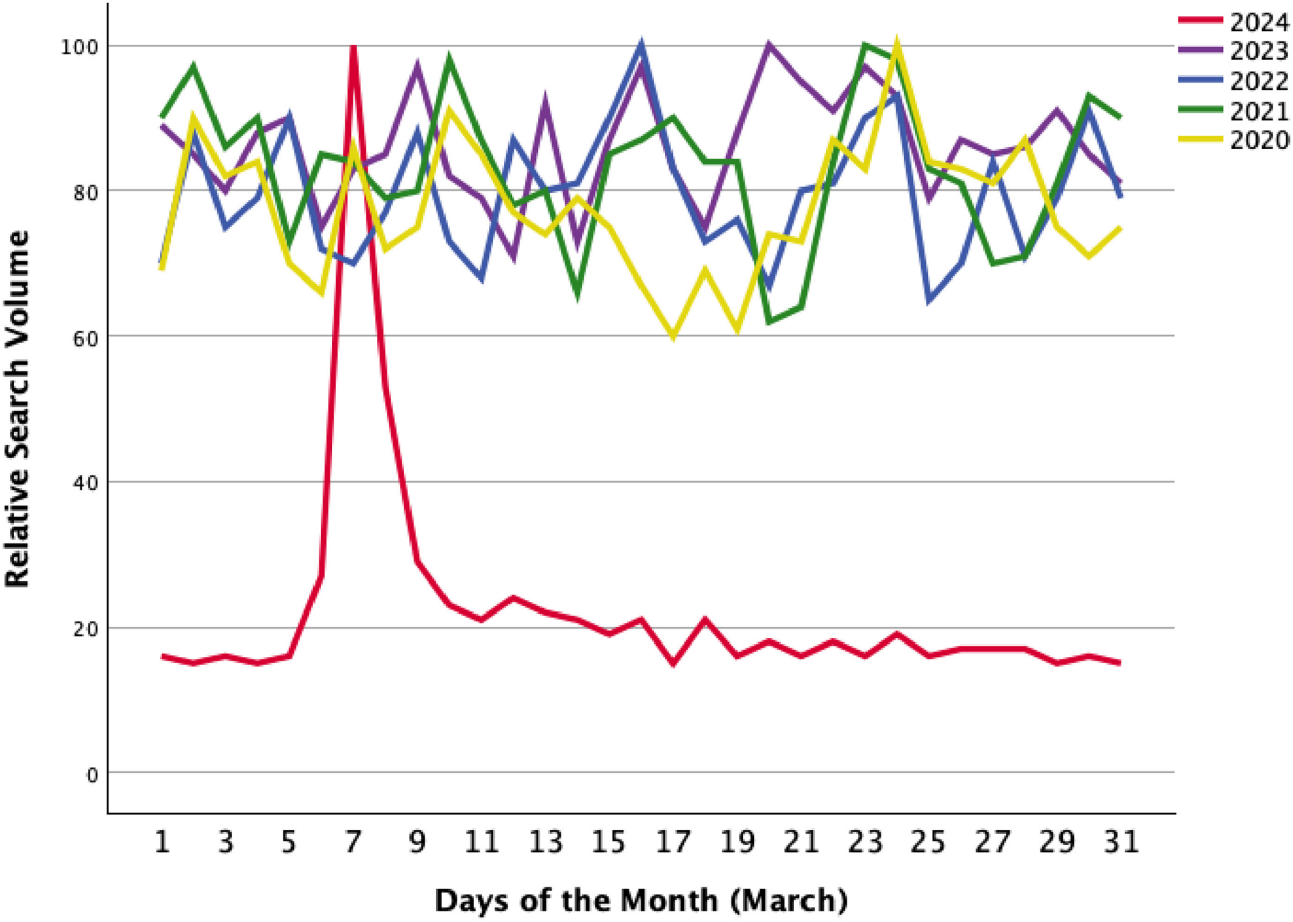
Relative search volume of search term “benzoyl peroxide” from March 2020–2024.

Our examination of Google Trends data post‐Valisure's FDA petition in March 2024 highlights nuanced shifts in public interest regarding BPO products, providing insights into consumer perceptions amidst safety concerns and the interplay between these concerns and consumer behavior.

Our study is limited by the absence of RSV data for specific BPO‐related search terms, including brand names of products containing BPO from 2020 to 2024, requiring a focus on complete datasets to maintain consistency. Furthermore, Google Trends analysis lacks the ability to access RSV data from other search engines and cannot subset data by subpopulations, offering only population‐level trends.

Dermatologists and healthcare practitioners need to adapt communication strategies and treatment approaches to address patient concerns effectively, staying vigilant to changing perceptions and navigating evolving safety considerations in dermatological practice.

## CONFLICT OF INTEREST STATEMENT

None declared.

## Data Availability

Not Applicable.
